# Non-Compliance and Follow-Up in Swedish Official and Private Animal Welfare Control of Dairy Cows

**DOI:** 10.3390/ani8050072

**Published:** 2018-05-08

**Authors:** Frida Lundmark Hedman, Jan Hultgren, Helena Röcklinsberg, Birgitta Wahlberg, Charlotte Berg

**Affiliations:** 1 Department of Animal Environment and Health, Swedish University of Agricultural Sciences, Box 234, SE-53223 Skara, Sweden; jan.hultgren@slu.se (J.H.); lotta.berg@slu.se (C.B.); 2 Department of Animal Environment and Health, Swedish University of Agricultural Sciences, Box 7068, SE-75007 Uppsala, Sweden; helena.rocklinsberg@slu.se; 3 Department of Law, Åbo Akademi University, Gezeliusgatan 2, 20500 Åbo, Finland; birgitta.wahlberg@abo.fi

**Keywords:** animal welfare, audit, compliance, dairy cow, policy making, quality assurance scheme

## Abstract

**Simple Summary:**

In many cases, different animal welfare inspections are taking place at an animal farm over time, as the farmer has to comply with both the legislation and with various private standards. In this study, we compared official inspections carried out by CAB (the County Administrative Board, a governmental agency) with private inspections carried out by Arla Foods (a private company) on dairy farms in one Swedish county. For example, we looked at seasonal effects and compared the incidence of different non-compliances. This study shows that long time periods were sometimes allowed for correction, that follow-up systems are diverse, and that there were differences in the inspection result between CAB and Arla due to different focuses during the inspections. Dirty dairy cattle were, however, a common non-compliance found by both CAB and Arla. Tie-stall housing and winter season (Dec–Feb) were risk factors for non-compliance, while the risk was lower for both CAB and Arla to find non-compliances at organic farms compared to conventional farms. We conclude that the presence of both similarities and differences between different control systems underlines the need for transparency, predictability, and clarity of inspections.

**Abstract:**

Farmers often have to comply with several sets of animal welfare regulations, since private standards have been developed in addition to legislation. Using an epidemiological approach, we analysed protocols from animal welfare inspections carried out in Swedish dairy herds by the County Administrative Board (CAB; official control of legislation) and by the dairy company Arla Foods (private control of Arlagården standard) during 2010–2013 in the county of Västra Götaland. CAB and Arla inspections were not carried out simultaneously. We aimed to identify common non-compliances, quantify risk factors of non-compliance, and investigate if non-compliances were based on animal-, resource-, or management-based requirements, as well as determining the time period allowed for achieving compliance. Non-compliance was found in 58% of CAB cases, and 51% of Arla cases (each case comprising a sequence of one or several inspections). Dirty dairy cattle was one of the most frequent non-compliances in both control systems. However, the differences in control results were large, suggesting a difference in focus between the two systems. Tie-stall housing and winter season (Dec–Feb) were common risk factors for non-compliance, and overall organic farms had a lower predicted number of non-compliances compared to conventional farms. The presence of both similarities and differences between the systems underlines the need for transparency, predictability, and clarity of inspections.

## 1. Introduction

The majority of European citizens believe that the welfare of farm animals should be protected [1]. The goal of protecting animals is evidently based on an increase of political activity nationally and within the EU, with the adoption of legislation and action plans promoting the protection and welfare of animals [2]. Animal welfare concerns have also led to the development of private initiatives by non-government actors [3], for example, private standards initiated by different stakeholders in the food chain [4]. Farmers can choose, more or less voluntarily, to certify their production according to different kinds of private standards [4,5,6,7]. Therefore, in practice, a farmer has to comply with several different animal welfare regulations and hence receive several types of inspections. Previous studies have demonstrated a trend towards an increasing number of inspections from various authorities and private bodies [8].

Both legislation and private standards can improve animal welfare to some extent, provided they are effectively implemented and enforced [9]. Furthermore, there has to be a control system connected to a regulation; including measures used for ensuring compliance, inspection frequencies, enforcement decisions allowed when non-compliance is discovered, time periods given to farmers to make corrections, and strategies and methods to follow up non-compliances. Otherwise, it is hard to claim anything about the actual effect of a regulation on the animal welfare in a country or region. To change people’s behavior and way of keeping animals accordingly with the legislation in force, the authorities must also interpret the legislation and handle their enforcement decisions in a respectful and administratively correct way, in order to ensure transparency and legal security for the farmers.

To ensure that official controls are trustworthy, EU legislation requires that Member States engage a competent authority to carry out the official animal welfare control impartially and effectively (Regulation No. 882/2004 of the European Parliament and of the Council on official controls performed to ensure the verification of compliance with feed and food law, animal health and animal welfare rules; hereafter Reg. 882/2004/EU). The European Commission’s inspection service Health and Food, Audits and Analysis (previously known as the Food and Veterinary Office, FVO) regularly audits control systems in all Member States [2]. In response to the increasing number of private standards, the EU has also developed best practice guidelines for private standards to improve their transparency, credibility, and effectiveness [10]). In order for a private standard to be trustworthy, compliance must be verified through reliable inspections at the farm level, including systems for handling non-compliances [11].

During recent years there has also been a discussion about the way to regulate animal welfare. A mixture of resource-, management-, and animal-based requirements and measures can be used in animal welfare regulations [12]. Legislation traditionally consists of mainly resource- and management-based requirements because of its preventive purpose, i.e., to reduce welfare risks [13]. Although these kinds of requirements are easier to assess, have a high inter- and intra-observer repeatability, and require little assessor training compared to animal-based requirements [14], several stakeholders, such as the European Commission, governments, and researchers have requested more animal-based than resource- and management-based requirements [14,15,16]. According to the European Food Safety Authority (EFSA) [17], the way in which a requirement is written will determine what type of measure (i.e., management-, resource- or animal-based) to use during an on-farm inspection to assess the level of compliance. However, in a previous study we concluded that this is not necessarily the case in practice [12]. Instead, the corresponding control guidelines often contain a wider spectrum of measures. For example, a resource-based requirement could also be assessed using animal- and management-based measures, reflecting the complexity of regulations [12]. The private standards in that study [12] focused somewhat more on assessing animal welfare at the farm level (in addition to prevention) compared to the legislation. This was not completely unexpected since the private standards often aim to assure good animal welfare to consumers and not merely reduce welfare risks [7], while the EU legislation for example mainly gives the minimum level of protection to ensure the minimum level of animal welfare.

Reg. 882/2004/EU states that the inspection frequency at farm level must be risk-based, i.e., farms with a higher risk for non-compliance and poor welfare shall be inspected more often. However, only a few studies have analysed risk factors for non-compliance, and hence knowledge is limited when it comes to identifying risk factors and prioritize between farms. It is also important to evaluate whether private controls contribute to the identification and rectification of relevant animal welfare non-compliances since affiliation to private standards may be included in the official animal welfare risk assessment system, thus subsequently influencing the frequency of on-farm inspections. This includes systems for achieving compliance, such as how advice is given, penalties, and efficient follow-ups. The regulations analysed in this article are Swedish, but the results may also be valid to other regions where private animal welfare standards are used as a complement to legislation and governmental control, which is often the case also in other European countries and to an increasing extent also in other parts of the world.

### Aim

Our main aims were to (1) identify common non-compliances during official animal welfare inspections and the private Arla audits, (2) quantify risk factors influencing the probability and level of non-compliance, and (3) determine the extent to which non-compliances were related to either an animal-, resource-, or management-based requirement. We also wanted to compare compliance with legislation amongst members of the different private standards Arlagården, Seal of Quality and KRAV. We hypothesized that most non-compliances would be resource- or management-based for all regulations, that the proportion of animal-based issues would be higher for private standards than for legislation, and that membership in a private standard would decrease the risk of non-compliance.

An additional aim was to investigate the time period that farmers were given for rectifying Arlagården and legislative non-compliance, and the number of follow-up inspections required before full compliance was reached. Our hypothesis was that the regional County Administrative Boards (CAB) carry out a larger number of on-farm follow-up inspections, compared to private standard organisations.

## 2. Materials and Methods

### 2.1. Terminology

In this paper, ‘legislation’ refers to the legal system and the legally binding regulation, i.e., the written law and the decrees concerning animal welfare and protection created by the state. ‘Standard’ refers to all other kinds of regulatory systems, like assurance schemes, animal welfare programmes, policies, certification schemes, etc. We use the term ‘regulations’ in reference to both legislation and standards. The term ‘control’ and ‘inspection’ are used synonymously, covering both official and private inspections and audits, in accordance with Reg. 882/2004/EU. A ‘control case’ (or ‘case’, simply) in this paper refers to an inspection or a sequence of inspections made at a farm, from the first inspection when the case was initiated to the last follow-up inspection when compliance should have been recorded, i.e., an episode of non-compliance. Furthermore, we use the term ‘inspector’ for the person carrying out such inspections, regardless of whether that person was employed by the official authorities, by a private standard organisation, or by a third-party audit provider.

### 2.2. Dairy Cow Welfare Control in Sweden

The competent authority that conducts official animal welfare controls at the farm level in Sweden is the CAB [18]. There are several different types of on-farm official CAB controls. Planned inspections may be risk-based, thematic, i.e., related to a specific matter such as the housing of dairy calves or dairy cow summer pasture access, or random [19]. There are also planned cross-compliance inspections linked to direct EU subsidies to farmers [19]. In contrast, inspections based on public complaints regarding abuse and neglect of animals are less predictable. The frequencies of risk-based and cross-compliance inspections depend on a risk classification system (Reg. 882/2004/EU). At least 10% of all Swedish farm animal premises are inspected each year, in accordance with the official Control Plan [20], and at least 50% of these controls should be planned inspections (SJVFS 2008:67). Until 2014, CAB inspections were normally not notified in advance. Since 2014, inspections motivated by complaints are still never notified, whereas planned inspections may sometimes be notified no more than 24 h prior the inspection. 

All dairy farmers delivering milk to the dairy company Arla Foods AB must comply with the Arlagården standard. Arla is the biggest dairy processor in Sweden and controls approximately 50% of the market for Swedish dairy products [21]. Since Arla is an international company, Arlagården also operates in other European countries. Arlagården’s inspectors in Sweden are hired by Arla from the advisory organisation Växa Sweden (Bernt Andersson, Arla Foods AB, pers. comm. 2015-02-15). Farmers in Sweden delivering milk to other dairy processors can or must (depending on the dairy company’s policy) comply with the requirements in the standard Seal of Quality, and if farmers choose to deliver organic milk they also have to comply with the requirements in the Swedish organic standard KRAV [7]. Seal of Quality and KRAV hire independent certification bodies, authorized by the Swedish Board for Accreditation and Conformity Assessment (Swedac), to audit farms [22,23]. Control frequencies are approximately once a year (KRAV), every two years (Seal of Quality), and every three years (Arla). Private audits are normally notified well in advance.

### 2.3. Data Collection

Two separate sets of data were collected by using records from official animal welfare inspections conducted by both CAB and Arla on dairy farms in Västra Götaland county in south-western Sweden during 2010–2013. We restricted this study to one county to ensure that farms were inspected under both regulations while limiting the amount of data and the number of inspectors involved. There are numerous dairy farms in Västra Götaland although the numbers have declined from 1019 in 2010 to 828 in 2013 [24]. Inspection reports based on legislation (CAB) or Arla’s scheme were the main documents analysed, but also other documents and decisions were collected and analysed, i.e., prohibitions and orders (hereafter ‘injunctions’, decided by CAB), prohibitions to keep animals (CAB), decisions to seize animals (CAB), and temporary blocking of milk delivery (Arla). Photocopies of CAB documents were provided by mail. Arla documents were analysed on-line at the Arla Foods AB office in Jönköping.

Based on the retrieved information, the following variables were formed at the case level; control system (CAB or Arla), year (2010, 2011, 2012, or 2013), season (Mar–May, Jun–Aug., Sep–Nov, or Dec–Feb), control type (CAB: complaint, risk-based, random, thematic pasture, thematic other, cross-compliance, or not defined; Arla: start-up, planned, or follow-up), identity of inspectors (*n* = 76 for CAB, *n* = 11 for Arla), cow housing system (cubicles (free-stalls), tie-stalls (tethered cows), or mixed), Arla member (yes or no); KRAV member for organic production (yes or no); Seal of Quality member (yes or no), notification (whether the inspection was unannounced) (yes or no); herd size (number of dairy cows), number of non-compliances, types of non-compliance (categorized as dirty cattle, inadequate housing of calves (e.g., inadequate design of pens, tied-up, or kept in single pens when overage), wet or dirty lying areas, overstocking (i.e., too little space available), inadequate feed and water supply (e.g., insufficient access or poor quality), high risk of injuries, lack of necessary animal facilities (e.g., none or insufficient number of sick or calving pens), poor floor condition (e.g., slippery, broken, too large a proportion of slatted floors), not enough time on pasture, thin animals, overgrown claws, unclipped cattle, ventilation alarm system missing, lack of supervision and care (e.g., untreated sick animals that were not inspected daily, staff not sufficiently competent or animals in need of re-grouping), dirty cowsheds, or other (e.g., poor air quality, insufficient light in barns, and loud mechanical noise), types of decision taken or document established by CAB or Arla based on the inspection (inspection report, injunction, prohibition to keep animals, seizure of animals or prohibition of milk delivery); time between non-compliance and correction; number of follow-up inspections (as motivated by non-compliance); type of follow-up inspections (on-farm or administrative); and whether compliance was reached before the case was closed (yes or no). An administrative CAB inspection meant that the farmer demonstrated compliance by providing photos, receipts, veterinary certificates, etc., without a farm visit. For an administrative Arla inspection, the farmer him/herself guaranteed that the necessary corrections had been made by signing and returning a certificate to Arla.

When information about affiliation to private standards was not provided in the CAB reports we contacted Arla, KRAV, Seal of Quality or the milk processor plants in order to receive this information.

### 2.4. Data Editing and Analysis

The data were entered into Excel 2013 (Microsoft Corp., Redmond, WA, USA) and they were edited and analysed using StataIC 13 (StataCorp, College Station, TX, USA). Two dependent variables were constructed, one expressing whether or not non-compliances were found (0, full compliance; 1, one or more non-compliance) and the other expressing the number of non-compliances found.

The frequency of non-compliance was analysed for CAB and Arla controls separately, using control case as the unit of analysis. Multilevel mixed-effects logistic regression was applied to model the probability of one or more non-compliance in a control case. In addition, multilevel mixed-effects negative binomial regression was used to model the number of non-compliances per case. In all four models, farm identity was included as a random effect to account for clustering on farms.

Fixed effects offered for inclusion in the models were year, season, control type, housing, Arla affiliation (CAB models only), KRAV affiliation, prior notification, herd size, and herd size squared. Initially, these independent variables were tested in univariable models. Herd size was tested together with herd size squared to check for a curvilinear relationship, and then alone. Only effects that were univariably significant at *p* ≤ 0.20 were considered eligible for further analysis. Multivariable models were constructed by backward stepwise elimination, retaining effects that were statistically significant at *p* ≤ 0.05, or effects that modified the coefficients of other covariates by more than 10%, indicating confounding. Already eliminated variables were tested for re-entry into the model at each step. Two-way interactions between significant main effects were assessed and retained if statistically significant (*p* ≤ 0.05). Model diagnostics performed included the link test [25] to identify model specification error, and examination of tolerance to assess collinearity. Model estimates were converted into odds ratios (OR; logistic models) and incidence risk ratios (IRR; negative binomial models). In the final models, differences with the reference level at *p* ≤ 0.05 were regarded as significant.

The effect of different Arla inspectors was estimated by constructing models of the probability of one or more non-compliance and the number of non-compliances, including a random inspector effect but no fixed effects and disregarding possible clustering by farm, using the latent variable approach [26] for variance partitioning, assuming a binomial distribution. The effect of CAB inspectors could not be estimated due to the large number of inspectors involved; often more than one being involved in the same inspection and up to nine in the same case.

Chi-squared analysis was performed in Minitab 16 (Minitab Inc., State College, PA, USA) to test differences in types of non-compliance during official controls between KRAV (organic) and non-organic conventional farms and between Arla and Seal of Quality farms, respectively, using case as the unit of analysis.

## 3. Results

### 3.1. Number of Farms and Cases

The number of farms inspected was similar between CAB (458) and Arla (472) (Table 1). Out of the 328 farms that were inspected by both control bodies during the four-year study period, 47% were inspected by both CAB and Arla within the same 6-month period and 14% of the farms were subject to CAB and Arla cases at the same time.

The number of cases per farm ranged from 1 to 7 with an average of 1.3 for CAB and from 1 to 4 with a mean of 1.4 for Arla. A larger number of inspectors were involved in the official CAB inspections than in Arla inspections, and CAB had a slightly higher proportion of cases with non-compliance than Arla (Table 1). However, an inspection rarely had other consequences than an inspection report summarising the results of the inspection and listing the non-compliances, if any. Only 9% of the CAB cases with non-compliance led to injunctions, 19% of these being combined with a conditional fine. Decisions to seize dairy cattle were made at two farms, but in the end none of the farmers was prohibited from keeping dairy cattle. As a result of the Arla inspections, eight farms (2%) were temporarily barred from delivering milk to the processing plant until compliant.

### 3.2. Types of Non-Compliance

**Figure 1:**
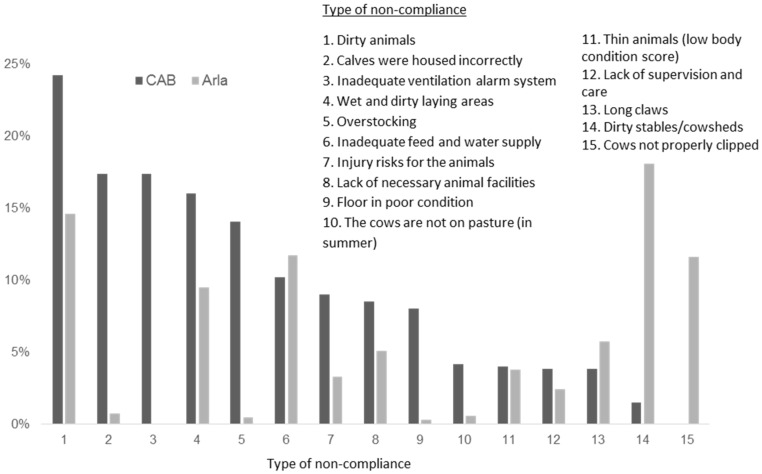
The proportion of control cases related to certain types of non-compliance. Based on data from the official animal welfare control (CAB = County Administrative Board) and the audits made by Arlagården private standard (Arla) on the private standard Arlagården in the Swedish county of Västra Götaland during 2010–2013.

The presence of dirty cattle was the most common non-compliance in the CAB control and the second most common in the Arlagården standard (Figure 1). Arla inspectors also found evidence of unclipped animals (farmers in Sweden normally clip the coats of the cows during winter), a requirement which was not included in the legislation and hence not checked by CAB inspectors. The legislation, on the other hand, covered ventilation alarm systems, which Arla did not include.

Compared to CAB controls, Arla inspections revealed a higher proportion of non-compliance related to dirty cowsheds, inadequate feed and water supply, and overgrown claws (Figure 1). The CAB controls did, on the other hand, in addition to dirty animals, involve more non-compliances related to the housing of calves, overstocking, floors in poor condition, and inadequate time on pasture.

There was no effect on the type of non-compliance found during CAB control by private standard affiliation, i.e., KRAV vs. conventional (Chi-Square (12 df); *p* = 0.86), and Seal of Quality vs. Arla (Chi-Square (12 df); *p* = 0.58).

Twenty-seven percent of all Arla cases and 53% of Arla’s cases with deficiencies had non-compliances concerning animal-based requirements (e.g., too dirty or thin animals, overgrown claws, untreated sick or injured animals); the corresponding numbers for CAB were 27% (of all cases) and 47% (of cases with remarks). Fifty-four percent of all CAB cases, and 93% of the CAB cases with remarks, had non-compliances related to resources or management. The corresponding numbers for Arla were 39% (of all cases), or 77% (of cases with remarks).

### 3.3. Risk Factors for Non-Compliance

The complete multivariable models are presented in Table 2 and Table 3. The highest risk for both one or more non-compliances and a higher number of non-compliances was the presence of tie-stall housing, both for CAB and Arla cases. The odds were 2.58 times higher (*p* = 0.001) to find any non-compliance in a CAB case with tie-stalls than in cubicle housing (Table 2).

The probability of a non-compliance was lower at a CAB pasture related thematic inspection or a cross-compliance inspection, compared to a random inspection (Table 2 and Table 3). The odds were 2.16 times higher (*p* = 0.047) to find one or more non-compliances if the farmer was notified prior to a CAB inspection (Table 2). However, by an alternative modelling approach, notification did not have any effect on the number of non-compliances (Table 3). Farmers received prior notification in 11% of CAB cases while Arla routinely notified the farmer before an inspection.

Non-compliance was more common during winter (Dec–Feb) in both CAB and Arla cases, compared to summer (Jun–Aug) (Table 2 and Table 3). There was a lower probability (OR = 0.46, *p* = 0.006) for finding one or more non-compliances at a KRAV farm during an Arla audit (Table 2). The incidence of non-compliance was also lower when inspecting a KRAV farm both in CAB (IRR = 0.53, *p* = 0.001) and Arla cases (IRR = 0.51, *p* = 0.038), compared to a conventional farm (Table 3). However, due to interaction, there was an effect of KRAV affiliation on the incidence of non-compliance in Arla cases only in summer, autumn, and winter.

The identity of inspectors had a significant effect on both the probability of compliance (*p* = 0.0009) and the number of non-compliances (*p* < 0.0001) in an Arla case, although only 3.1% of the variation in non-compliance was attributable to differences between inspectors, according to the intraclass correlation coefficient, indicating that the variation between inspectors was limited.

The herd size in this study varied from 1 to 500 dairy cows, with a median of 45. However, neither ‘herd size’ nor ‘year’ was related to CAB or Arla inspection results (joint Chi-square *p* > 0.05) so these variables were not included in the final models. Nor was ‘Arla affiliation’ included in final CAB models; compliance did not depend on affiliation to Arla or Seal of Quality (*p* > 0.05).

### 3.4. Time Periods for Correction and Follow-Up Inspections

In general, farmers were given more time to correct non-compliances by CAB inspectors compared to Arla inspectors (Table 4). Arla inspectors always specified an exact timeline while CAB inspectors were less precise. Some of these expressions were possible to convert into exact periods (e.g., ‘a month’ translated to 30 days and ‘before autumn’ could be interpreted as number of days to September 1st); however, some of the deadlines, such as ‘as soon as possible’ or ‘at next planned inspection’ were impossible to convert to exact time. It was also quite common for CAB inspectors not to provide a deadline (Table 4).

CAB carried out additional follow-up inspections in 42% of all the cases, and Arla in 45%. Most commonly, both CAB and Arla made only one follow-up inspection when non-compliances were observed (Table 5). In 27% of the cases with non-compliances, CAB did not carry out any follow-up inspections, the corresponding number for Arla was 2%. At most, twelve follow-up inspections were made in one CAB case; the corresponding number for Arla was six. CAB performed follow-up on-farm inspections in 44% of the cases with issues; the corresponding number for Arla was 28%. Arla performed administrative follow-up inspections in 64% of the cases of non-compliance, while the CAB performed such inspections in 39% of cases.

Follow-up inspections did not necessarily mean that complete compliance was reached. Not all non-compliances were always checked during follow-up inspections and some cases were closed despite remaining non-compliances. Thirty percent of the CAB cases (Arla 0%) had remaining non-compliances when they were closed and 31% of the CAB cases (Arla 11%) had non-compliances that ‘disappeared’ during the handling of cases (i.e., the outcome of the non-compliance was not recorded). In some cases, follow-up inspections had not yet been carried out in Feb 2015 (i.e., last time data collection for this study was made). Of the CAB cases 42% were closed when total compliance was reached and documented; the corresponding number for Arla was 89%.

The percentage of farms that achieved compliance within the given time frame did not differ and was not dependent on whether the non-compliance was resource-, management-, or animal-based (Table 6). However, non-compliance resulting from too many thin animals was often associated with more than one follow-up inspection in both CAB and Arla controls, as was non-compliance regarding missing ventilation alarm system in CAB (29% of non-compliances). 

## 4. Discussion

Regulatory requirements undoubtedly have a normative function, but they are likely to remain rather hollow instruments if not implemented and enforced. The main purpose of an inspection is to assess whether compliance is reached to the acceptable minimum level within the areas covered by the regulation. For example, lameness and mastitis are severe welfare problems for dairy cows [9,27], but such problems are not necessarily covered by animal welfare regulations. Only unattended or untreated disease or injury was considered as regulatory non-compliance; if a sick animal was found to be under relevant treatment, it was usually not recorded.

It is also important to stress that this type of study does not cover factors related to the actual on-site meeting and dialogue between farmers and inspectors. Other studies suggest that, for example, personality, expectations, trust, gender inequality, and oral and body language may have an effect on the outcome of an inspection, especially if a regulation is open for interpretation [28,29].

Hence, control statistics alone do not provide a complete picture of the actual animal welfare level in a region or country, but it does indicate areas of concern and trends over time.

### 4.1. Types of Non-Compliance

According to the Federation of Swedish Farmers [15], Swedish farmers believe that the official control focuses more on resource-based technical details than the actual welfare of the animals. The Health and Food, Audits and Analysis (previously known as the Food and Veterinary Office, FVO), on the other hand, has criticized Sweden for taking too few resource-based measurements on farm [30]. Hence, different stakeholders have very different impressions and interpretations of what is happening during the inspections. According to our study, the most common non-compliance registered by CAB during the official inspections at dairy farms was related to animal welfare outcomes (dirty animals). The identification of dirty and soiled animals as the number one welfare problem in dairy herds in this study is worth emphasizing. The presence of dirty animals was also a common non-compliance in Arla, but not to the same extent. Cleanliness of dairy cows is an animal welfare concern since dirty cows are at increased risk of mastitis [31], may have difficulties to thermoregulate [27], and risk developing painful skin burns and dermatitis. Furthermore, poor cleanliness is a clear example of a problem where a combination of resource-based input measures related to housing system (e.g., design of cubicles (free-stalls)), management input (e.g., amount of clean bedding provided, frequency and thoroughness of cleaning the cow shed, and grooming the cows) and animal-based outcome measure (i.e., the actual cleanliness of the cows) is useful in identifying an animal welfare problem and its causes.

Arla’s most common animal welfare non-compliance was related to management of resources (dirty cowsheds, poor maintenance), which possibly results from a general focus on food hygiene. Also, the official control often identifies non-compliances that are resource- or management-based, since almost all cases with remarks had this kind of non-compliance, even if several cases also had animal-based non-compliances. Our results are in accordance with Wahlberg [32], who noted that the most common non-compliances in Finnish official animal welfare control were about management and resources. Also, in France, the official control focuses on resources and management [33]. This is not surprising considering the current EU legislation. Sweden has deliberately added animal-based requirements to the legislation and measures to the checklist used by official control [12]. It is important to be aware of such differences between countries before drawing conclusions about compliance or not with the animal welfare legislation, both regarding the number and the type of non-compliance. Our findings illustrate that animal welfare inspectors can assess both animal welfare risks and animal welfare outcomes, but that there will probably be differences between countries and regulations since the measures can differ even if the requirements seem to be quite the same at a first glance.

The CAB detected more non-compliances related to overstocking and other remarks concerning animal facilities, probably because the Arlagården standard covered fewer resource-based requirements. In line with this, we expected that Arla would find a higher proportion of non-compliances related to animal-based outcomes than CAB [12]. However, the proportion of registered animal-based non-compliances was quite similar between CAB and Arla, with CAB having a higher proportion of remarks on dirty animals and Arla on poor claw conditions. Växa Sweden, which carries out the inspections for Arlagården, is actively working on improving claw health and has hence put extra emphasis on this problem [34].

Another reason why the type of non-compliances to some extent may differ between CAB and Arla was the routine for notification of inspections. Some of the non-compliances are easy to correct when the farmer is given only a short notice, while others (for example excessively thin animals) cannot easily be resolved. However, the higher risk for non-compliances when CAB inspections had been notified in advance was quite surprising. We suggest that the explanation may be related to the situations in which the CAB choses to notify or not. According to the CAB, a notification was issued predominantly when practical problems at the inspection were expected, which means that the group of farms receiving a notification prior to the inspection was not randomly selected. There may have been a known history of non-compliances or avoidance of inspections by farmers’ absence, possibly with a more negative attitude towards animal welfare.

### 4.2. Differences in Approach between Regulations and Inspectors

One possible explanation for the relatively large proportion of dirty animals according to CAB is the legislation’s focus on protecting individual animals, versus Arla which assesses cleanliness at the group level [35]. In our study, assessment at group level was occasionally also used during official controls. CAB inspectors sometimes argued that a single dirty animal was not a systematic problem and therefore not a non-compliance. There is a general calibration problem when policy-making bodies establish different limits for comparable requirements, because this may result in different animal welfare levels in practice [36], and it is especially important for private policy makers to not consider their private standard as a single stand-alone phenomenon as a farmer will always need to comply with the binding regulation [37]. In addition, intra-observer agreement can be difficult to reach when there is no reference observer [38], or when there is no validated standard protocol to use when assessing a welfare parameter [39]. Recent studies have indicated problems with inter-observer agreement between official animal welfare inspectors [33,40,41], which may be the case also for private inspectors. In our study there was a difference in control outcomes depending on which Arla inspector conducted the inspection, even though Arla actively tried to improve inter-observer agreement (Bernt Andersson, Arla Foods AB, pers. comm. 2016-02-19). We do not believe that the large number of CAB inspectors with diverse backgrounds and the sometimes imprecise guidelines, resulted in more standardised assessments than Arla, rather the opposite.

The above mentioned frequent mixture and complexity of resource-, management-, and animal-based requirements and measures was evident also in this study. In the CAB inspection reports non-compliance was seldom explained and motivated from only one point of view. On the contrary, it was commonly described as a combination of several problems. For example, dirty animals were not always identified as an animal-based problem only, but also as management-based through poorly cleaned lying areas and resource-based through insufficient amounts of straw or poor housing. Furthermore, the resource- and management-based non-compliances were quite often motivated and explained on the basis of alleged negative effects on the animals if corrections were not made, i.e., the risk of poor animal welfare outcomes was explicitly mentioned. This is in line with the intentions of the legislation that aims to prevent poor welfare for each individual animal [7,13].

The regulatory requirements may change over time, which may temporarily affect the level of compliance. In this study, the Arlagården requirement relating to water access for calves was changed in January 2012, from water at least twice a day to ad libitum (free) access. Not surprisingly, there was an increase in this non-compliance after January 2012, which explains why Arla had more remarks than CAB on problems with feed and water access. Also, a change in the guidelines, i.e., in relation to the attention different requirements should be given and how to assess compliance, can have an impact. For example, according to Arla’s own statistics, the number of non-compliances related to animal cleanliness, body condition, and claw health more than doubled in a 10-year period due to a shift in focus, guidelines, and education of inspectors, and not due to any changes in the requirements per se.

### 4.3. Risk Factors for Non-Compliance

The percentage of organic dairy farms in Västra Götaland county increased during the course of this study from 10.5% in 2010 to 17.0% in 2013 [24]. Our result that the probability of finding non-compliance is lower at organic farms compared to conventional farms contradicts a previous study which found that organic farms performed poorly when compared to conventional farms during official controls in Sweden [42]. However, our result is consistent with KilBride and co-authors [43] who reported that organic farms were more likely to be compliant with animal welfare legislation in general. There are at least three possible reasons why the organic farms in our study had a higher level of compliance; (1) affiliation to an organic regulation is voluntary, which increases the probability of compliance as people who volunteer for things are more likely to comply with rules [44]; (2) organic farms received more inspections because they were affiliated with more than one standard, in addition to the legislation and Arlagården or Seal of Quality, and KRAV performed audits every year; (3) KRAV animal welfare standards were in some aspects stricter than the legislation and the Arlagården standard. Furthermore, inspector expectations and values may have an impact on the control result [29].

The higher probability of non-compliance in tie-stalls was not surprising and is in line with the reasons behind Sweden banning the construction of new tie-stall barns, a paragraph which came into effect in August 2007. Any such currently existing systems are relatively old. In 2015, approximately 32% of the Swedish dairy cows were still kept tied (Agneta Schultzberg, Växa Sweden, pers. comm. 2016-03-17). Nor was it surprising to find a higher probability of non-compliance during winter than in summer. Dairy cows are usually kept indoors during the winter but still develop a thicker coat, which makes it more difficult to keep them clean, if not clipped. The Swedish Animal Welfare Ordinance (SFS 1988:539) requires access to pasture for all cows during summer, based on overall animal welfare benefits [45,46], and the animals are then less likely to become very dirty. CAB carried out thematic inspections during summer, checking if animals were on pasture, but indoor facilities were usually not inspected at these occasions, which may have influenced the results. However, Arla also had a lower probability of non-compliance during summer, although they did not carry out thematic pasture inspections.

There are several possible explanations for the lower probability of non-compliance during a cross-compliance CAB inspection; (1) not all requirements were checked during cross-compliance inspections; (2) inspector knowledge that cross-compliance failure could lead to a cut in the economic subsidies to the farmer influenced the outcome of a cross-compliance inspection [41]; and (3) cross-compliance inspections in our study were not always carried out by regular trained animal welfare inspectors. Irrespective of the underlying reasons, it is important for CAB to discuss these differences in order to increase awareness among the inspectors.

Current EU legislation requires official controls to be risk based (Reg. 882/2004/EU), i.e., farms with a high probability of non-compliance shall be identified, and this information shall be used to allocate control resources for on-farm visits [13]. According to our study, type of housing system and time of year are relevant when designing a risk assessment tool. We found that affiliation to KRAV significantly decreased the animal welfare risks. However, all farms in this study were affiliated with at least one private standard; we could therefore not compare them to farms that were not affiliated to any private standard. Further studies of the influence of private standards on official control are therefore needed. KilBride and co-workers [43] and Clark and co-workers [47] reported that British farmers affiliated to private standards or other voluntary welfare schemes, organic or not, were more likely to comply with animal welfare legislation.

From an international perspective Swedish farms are relatively small. EFSA has defined a small-scale farming system as a herd size of less than 75 cows [48], in our study the median was 45 cows. In our study the herd size did not affect the probability for CAB or Arla to find non-compliances, showing that it may be more important to consider the type of housing system when assessing welfare risks. However, it would be interesting to see if similar studies carried out in countries with much larger farms than Sweden would render similar results.

### 4.4. The Use of Enforcement Decisions

Even if non-compliances were detected, CAB and Arla rarely made decisions about injunctions, prohibitions to keep animals, seizure of animals, or temporary blocking of milk delivery. Wahlberg [32] also found that enforcement decisions in Finnish official animal welfare control were rare. Our result indicates that most non-compliances were not seen as very severe by CAB and Arla, and that farmers were willing and good at making corrections. Repeated injunctions will, according to the Swedish Animal Welfare Act (1988:534), lead to a CAB order prohibiting the farmer from having animals, which could have made CAB reluctant to issue injunctions. In Sweden, as well as in the rest of the EU, the government authorities must consider the principle of proportionality when enforcing legislation, i.e., a legal measure must be appropriate, necessary, and balanced between asserting a public interest and the interference with the private interests concerned. Although Arla is not an official authority and Arlagården is a private standard and does not need to follow legislative texts that govern official control, they were restrictive in taking more enforcement decisions.

### 4.5. Time Periods for Correction and Follow-Up Inspections

The time provided for correcting non-compliances has not been previously analysed or described in the scientific literature. In the control guidelines to Arlagården, there are recommended time periods for different kinds of non-compliances, which correspond well to the median time periods for correction in our data. For CAB inspections there were no recommended time periods for correction. Arlagården used a template for the inspection report where inspectors must specify an exact time period, i.e., number of days, for each non-compliance, whereas the CAB used different templates with different options. Sometimes a specific deadline was set for each non-compliance and sometimes the CAB only indicated a date that would trigger a follow up inspection. This may explain why the same time period was sometimes given for rebuilding stables or trimming the animals’ claws. However, it may be argued that one deadline for the whole case including both urgent and less urgent or more extensive corrective actions is quite unrealistic and may lead to unnecessary delays of urgent actions.

Based on our study, Arla farms were recorded as compliant sooner than farms adhering to a CAB control. However, the quality and reliability of the different follow-up inspections may be questionable. In total, 64% of Arla’s follow-up inspections were administrative, i.e., the farmer was only asked to perform a self-assessment to prove that compliance had been reached. Of the CAB follow-up inspections, 40% were administrative. CAB, on the other hand, demanded some kind of independent proof of compliance, e.g., a photo or veterinary certificate. Lomellini-Dereclenne and co-workers [33] concluded that the proportion of fully compliant farms increased when farms were re-inspected. According to EU best practice guidelines for certification schemes an inspection should be carried out by an impartial body [10], i.e., not by self-assessment. Restricted use of self-assessments can be acceptable, but should not risk reducing the trustworthiness of the regulation [49].

CAB more often decided to close a case despite of unresolved non-compliances, often based on the inspector’s belief that the farmer would comply without any further inspections. In other cases, CAB would close an unresolved case to focus on the most urgent problems, i.e., cases with seriously reduced animal welfare, because CAB resources were, and still are, limited. According to Lipsky [28], the limitation of resources is a well-known problem for public authorities all over the world, regardless of country or region.

Both CAB and Arla had cases where the outcome of the non-compliance was not recorded. For Arla, there were mainly three reasons for this. Firstly, some results from administrative follow-up inspections in the beginning of 2010 were missing in the computer system. Secondly, farmers may have chosen to leave Arla for another dairy processor, and hence Arla were not able to carry out any follow-up inspections. Finally, farmers may have chosen to terminate milk production before Arla made a follow-up inspection. The two last reasons are interesting from an animal welfare point of view, because Arla would then cancel follow-up although there may still be some animals left on farm (non-lactating cows and other types of cattle, or lactating cows but milk delivered to another dairy processor). It is, inevitably, possible for a farmer to exit voluntary private standards. In contrast, legislation always applies.

The reasons why the outcomes of non-compliances were not always recorded during CAB control cases are not clear. Possible reasons include insufficient documentation during and after an inspection, difficulty of keeping track of non-compliances in large cases where several inspectors were involved, and problems with the case management computer system. The Swedish operational official animal welfare control was relocated from the municipalities to the CAB one year prior to the documentation used in this study, requiring establishment of new structures and routines. There may also be regional differences in how the control was handled between different CABs. However, from the point of view of both animal welfare and legal security it is crucial that all involved stakeholders are aware when compliance has been reached.

## 5. Conclusions

As a response to a growing public concern for farm animal welfare globally, one can see an increasing interest in private animal welfare standards in many countries. However, as this study shows, the audits of the standards constitute a complex area and we argue that it may be wise to remain cautious with respect to relying heavily on private standards. In most countries, their legal implications and relevance in relation to governmental legislation have still not been fully analysed from a legal perspective.

In this study, a private standard and the national legislation was compared, as the co-existence of several parallel control or audit systems is a rather common situation in most countries holding animal welfare legislation. Our result show that although the same farms were visited and inspected by both private and governmental inspectors, their audits differ at certain points of core relevance for animal welfare. In this case, the two audit systems, CAB and Arla, had almost the same proportion of animal-based non-compliances, where CAB recorded ‘dirty animals’ most often and Arla recorded ‘dirty cowsheds’ most often. However, most non-compliances found, by both control bodies, concerned non-animal-based measures where tie-stall housing and winter season were common risk factors for non-compliance. Affiliation to the private standard KRAV was associated with a decreased risk of non-compliance. The authors have no reason to believe that these findings are completely country specific; on the contrary, we hypothesize that a similar distribution of similarities and differences—although possibly for other factors—would be found also in other countries or regions.

The presence of both similarities and differences between the official CAB and private Arla control systems demonstrates the need for transparency, predictability, and clarity of inspections. It is reasonable to believe that attention to general administrative requirements such as the ones discussed here would be warranted also in other countries, especially since the development of private standards is increasing, and that both farmers and inspectors need to understand the prerequisites and purposes of the inspections being carried out. Similarly, to increase transparency for both farmers and citizens on the scope of inspection, clarification of perceived gaps between the overall aim of a certain regulation and the outcome of the inspections should be improved, and it should of course always be made very clear to the farmers receiving an animal welfare inspection visit (or any other type of audit) who is carrying out the inspection, in relation to which standard it is being carried out and on behalf of whom. For example, it might be useful to clarify why e.g., the presence of sick animals is not necessarily seen as a non-compliance (if, for example the animals are under veterinary treatment), or why the number of animals is regarded relevant in relation to whether a deficiency is to be seen as a non-compliance or not. In this regard, the legal justification of the decision is essential regardless of country or regulation, so that the farmers are given an opportunity to clearly understand what they must do and why, and before what date. We recommend dairy advisory services and veterinarians to increase focus on dairy cow cleanliness through improved housing system design, use of bedding material, clipping of animals, and general cleaning and hygiene routines. Further, to increase compliance but also transparency and accountability, we would recommend any auditing body—being it governmental or private—to state clear and not overly extended deadlines for correction of non-compliances, and to carry out relevant follow-up within reasonable time.

In general, our study shows that, based on the animal welfare inspection results, continued summer grazing and a ban on tie-stalls for dairy cows would have a positive impact on individual animal welfare. This conclusion, of course, applies to all countries where seasonal outdoor grazing systems and/or tie-stalls are still in practice.

## Figures and Tables

**Table 1:** Information about the number of farms, cases and inspectors, and prevalence of non-compliance, in the official animal welfare control (CAB = County Administrative Board) and Arlagården private standard (Arla) on dairy farms in the Swedish county of Västra Götaland during 2010–2013.

Background Aspect	CAB	Arla
Number of farms visited	458	472
Number of cases	599	665
Cases with non-compliance	347 (58%)	336 (51%)
Number of inspectors	76	11

**Table 2:** Final multivariable logistic regression model of the probability of one or more non-compliances found during a case of official (CAB = County Administrative Board) or private (Arla on the private standard Arlagården) animal welfare control in the Swedish county of Västra Götaland during 2010–2013.

Variable	CAB			Arla		
	OR	SE	*p*-Value	OR	SE	*p*-Value
Intercept	2.68	1.03	0.010	0.98	0.21	0.94
Season			0.023 ^1^			0.002 ^1^
*Winter (Dec–Feb)*	1			1		
*Spring (Mar–May)*	0.65	0.19	0.14	0.66	0.15	0.06
*Summer (Jun–Aug)*	0.23	0.12	0.005	0.31	0.10	<0.001
*Autumn (Sep–Nov)*	0.44	0.16	0.026	0.95	0.21	0.81
Control type			0.062 ^1^			
*Random*	1					
*Complaint*	0.45	0.19	0.062			
*Risk-based*	0.73	0.28	0.41			
*Thematic pasture*	0.28	0.18	0.046			
*Thematic other*	0.57	0.20	0.11			
*Cross-compliance*	0.12	0.08	0.002			
*Other*	0.64	0.67	0.67			
Notification			0.047 ^1^			
*No*	1					
*Yes*	2.16	0.84	0.047			
Housing			0.003 ^1^			0.008 ^1^
*Cubicles*	1			1		
*Tie-stalls*	2.58	0.72	0.001	1.79	0.37	0.005
*Mixed*	1.75	1.14	0.39	0.46	0.43	0.407
KRAV						0.006 ^1^
*No*				1		
*Yes*				0.46	0.13	0.006

^1^ Joint Chi-square test of effect.

**Table 3:** Final multivariable negative binomial regression model used to investigate the effect of different variables on the odds for a higher number of non-compliance during animal welfare control made by the CAB (County Administrative Board, official control) or Arla (on the private standard Arlagården) in the Swedish county of Västra Götaland during 2010–2013.

Variable	CAB			Arla		
	IRR	SE	*p*-Value	IRR	SE	*p*-Value
Intercept	1.56	0.30	0.019	0.77	0.11	0.071
Season			0.035 ^1^			<0.001 ^1^
*Winter (Dec–Feb)*	1			1		
*Spring (Mar–May)*	0.97	0.13	0.81	0.70	0.10	0.012
*Summer (Jun–Aug)*	0.46	0.12	0.004	0.44	0.10	<0.001
*Autumn (Sep–Nov)*	0.88	0.15	0.47	0.88	0.17	0.38
Control type			<0.001 ^1^			
*Random*	1					
*Complaint*	1.33	0.26	0.14			
*Risk-based*	1.12	0.19	0.50			
*Thematic pasture*	0.44	0.15	0.02			
*Thematic other*	0.90	0.14	0.52			
*Cross-compliance*	0.26	0.11	0.002			
*Others*	1.85	0.83	0.17			
Notification			0.28 ^1^			
*No*	1					
*Yes*	1.19	0.20	0.28			
Housing			0.035 ^1^			0.002 ^1^
*Cubicles*	1			1		
*Tie-stalls*	1.42	0.19	0.01	1.53	0.21	0.001
*Mixed*	1.15	0.40	0.69	0.39	0.31	0.23
KRAV			<0.001 ^1^			0.038 ^1^
*No*	1			1		
*Yes*	0.53	0.10	0.001	0.51	0.16	0.038
Season × KRAV						0.038 ^1^
*Winter* or *No*				1		
*Spring* × *Yes*				2.18	0.94	0.068
*Summer* × *Yes*				0.42	0.47	0.44
*Autumn* × *Yes*				0.60	0.31	0.31

^1^ Joint Chi-square test of effect.

**Table 4:** Time periods given for correction of the seven most common non-compliances found in both official animal welfare control (CAB = County Administrative Board) and Arlagården private standard audits (Arla) in the Swedish county of Västra Götaland during 2010–2013. The periods were calculated from the first time the specific non-compliance was observed.

Type of Non-Compliance	Median (Range) (Days)		as Soon as Possible ^1^	at Next Planned Inspection ^1^	No Deadline Given ^1^	Information Is Lacking or Ambiguous		Total Number of This Non-Compliance	
	CAB	Arla	CAB	CAB	CAB	CAB	Arla	CAB	Arla
Dirty animals	30 (6–300)	7 (1–60)	10%	2%	25%	2%	4%	150	97
Wet and dirty laying areas	30 (0–300)	8.5 (1–90)	9%	3%	23%	3%	7%	158	73
Inadequate feed and water supply	42.5 (0–195)	7 (1–240)	11%	0%	30%	0%	0%	82	91
Injury risks for the animals	42.5 (1–195)	30 (1–210)	13%	0%	18%	0%	0%	61	24
Lack of necessary animal facilities	90 (3–300)	90 (1–273)	0%	0%	21%	3%	0%	66	35
Thin animals	30 (1–210)	30 (2–45)	12%	4%	46%	8%	0%	26	26
Long claws	40 (7–210)	30 (7–45)	8%	8%	15%	4%	0%	26	38

^1^ Arla did always set a specific date for corrections so these categories were not applicable for Arla.

**Table 5:** Number of follow-up inspections to check for compliance after the first inspection of a case of non-compliance in official animal welfare control (CAB = County Administrative Board) and Arlagården private standard audits (Arla) in the Swedish county of Västra Götaland during 2010–2013. The sums are not 100% due to missing data.

Number of Follow-Up Inspections	Number (%) of Cases	
	CAB	Arla
0	95 (27.4)	6 (1.8)
1	170 (49.0)	272 (81.0)
2	65 (18.7)	21 (6.3)
3–4	11(3.2)	4 (1.2)
≥5	4 (1.2)	1 (0.3)
Data missing	2 (0.6)	32 (9.5)

**Table 6:** Total numbers of the seven most common non-compliances and percentages not corrected within the stipulated timeframe (more than one follow-up inspection carried out before compliance was registered) found in official animal welfare control (CAB = County Administrative Board) and Arlagården private standard audits (Arla) in the Swedish county of Västra Götaland during 2010–2013.

Type of Non-Compliance	Total Number of Non-Compliances		Number (%) of Con-Compliances Not Corrected within the Stipulated Timeframe	
	CAB	Arla	CAB	Arla
Dirty animals	150	97	27 (18)	1 (1)
Wet and dirty laying areas	158	73	36 (23)	0
Inadequate feed and water supply	82	90	18 (22)	5 (6)
Injury risks for the animals	61	24	7 (11)	2 (8)
Lack of necessary animal facilities	66	35	14 (21)	3 (9)
Thin animals	26	26	7 (27)	3 (12)
Long claws	26	38	5 (19)	3 (8)
